# Preparation of MnOx Supported LiOH Activated Soybean Oil Sludge Catalyst and Its Analysis in Denitration Mechanism of Selective Catalytic Oxidation (SCO)

**DOI:** 10.1038/s41598-019-47947-2

**Published:** 2019-08-12

**Authors:** Zhang Lei, Luo Min, Kong Tingting, Zhang Lei, He Huibin, Jia Yang, Yang Chao, Wu Yan, Li Mengting

**Affiliations:** 1 Xi’an University of Science and Technology, Xi’an, 710054 China; 2 Key Laboratory of Coal Resources Exploration and Comprehensive Utilization, Ministry of Land and Resources Xi’an, Xi’an, 710021 China; 3 Xi’an Shiyou University, Xi’an, 710065 China; 4 China National Heavy Machinery Research Institute, Xi’an, 710032 China; 5 Key Laboratory of Coal Resources Exploration and Comprehensive Utilization, Ministry of Land and Resources, Xi’an, 710054 China; 6 Neijiang Normal University, Neijiang, 641100 China

**Keywords:** Environmental chemistry, Materials for energy and catalysis

## Abstract

Treatment with selective catalytic oxidation (SCO) is an effective technology applied recently for conversion of nitrogen oxides pollution control. In order to solve the problems of high cost and difficulties in practical application of SCO catalyst, it was put forward using the solid waste sludge from soybean oil plant as catalyst carrier to prepare denitration catalyst. The sludge was treated by alkaline activation and then MnOx-based sludge was prepared by impregnation. Finally, MnOx-based sludge was calcined in the muffle furnace. The effects of activation and calcination conditions on catalyst activity were investigated. Fourier transform infrared (FTIR), X-ray photoelectron spectroscopy (XPS), X-ray diffraction (XRD) and scanning electron microscopy (SEM) were used to characterize the activity of the sludge based denitration catalyst, and the structure and activity of the sludge based denitration catalyst were furtherly confirmed. According to the achieved results, (1) after activated by LiOH with a mass concentration of 15% for 4 hours, the surface of the sludge catalyst has more alkali functional groups, making the denitration of sludge catalyst the best; (2) the MnOx-based catalyst calcined in the muffle furnace with calcination temperature of 450 °C for 4 hours has obvious denitration efficiency.

## Introduction

Nitrogen oxides have an increasing impact on air pollution. Two nitrogen oxides removal approaches such as selective catalytic reduction (SCR) and selective catalytic oxidation (SCO) have been applied to eliminate those Nitrogen oxides from polluted air flows. SCR has been widely used in developed countries because of its strong practicability, good selectivity and high conversion rate of product N_2_. But the method of SCR does have such problems as high cost of catalysts, highly reactive temperature and reductants needed to be added in the process of treatment. These restrictions increase the investment in the system and reduce the service life of the catalyst^1^. In recent years, SCO has been applied globally as a modern technology for air pollution control. The SCO process increases the service life of the catalyst and reduces the cost because the required temperature is low and there is no need to add oxidants. It is the key to select an appropriate catalyst during SCO process research. Sludge has abundant pore structure and sufficient specific surface area. Sludge as a carrier for prepared catalysts makes the loaded active ingredient work at full capacity. Using sludge as a catalyst in SCO process can not only improve the polluted air but also reduce the cost. As a refractory waste, sludge has potential research value and wide application prospect in the removal of nitrogen oxides with the environmental protection concept of using waste to treat waste. The atomic ratio of carbon to nitrogen (C/N) is an important index of soybean oil sludge and municipal sewage sludge. The carbon to nitrogen atomic ratio (C/N) of soybean oil sludge is 9.64 while the carbon to nitrogen atomic ratio (C/N) of municipal sewage sludge is within the range of 10–20^2,3^. According to previous studies, the smaller the carbon to nitrogen atomic ratio (C/N) of the sludge catalyst is, the greater the effect of removing nitrogen oxides is^4^. Not only the carbon to nitrogen atomic ratio (C/N) of soybean oil sludge but also its carbon content are lower than that of municipal sewage sludge. Lower carbon content can avoid the ignition of catalysts and reduce the influence of temperature on catalyst. Heavy metal content of more than 50 metals in soybean oil sludge was detected by ICP (Inductive Coupled Plasma Emission Spectrometer)^5^. These metal oxides can improve the catalytic activity of soybean oil sludge catalyst. Soybean Oil sludge is a good choice for the catalyst of SCO to remove nitrogen oxides. In the preliminary study of the research group, the activity of sludge catalyst can be improved much better by alkali activated than by acid activated. The structure, physical and chemical properties of sludge can be changed by alkaline activation. The metal loaded on the catalyst determines the cost of the catalyst. MnOx is cheaper than other rare earth elements and precious metals. Therefore, MnOx loaded catalysts have more research significance for the application of the SCO. This study originally proposed to use soybean oil sludge as the denitration catalyst carrier and carry out the alkaline activation treatment of the sludge and then use the impregnation method to prepare the MnOx supported catalyst. It is necessary to analyze the surface physicochemical characteristics of catalyst and investigate the influences of various factors on the removal rate of nitrogen oxides to achieve the optimum conditions for processes.

## Materials and Methods

### The pretreatment of sludge

The experimental raw material for this study was the dried soybean oil sludge from the Xi’an Bangqi Oil Technology Company Wastewater Treatment Station. Firstly the air-drying original sludge (soybean oil sludge) was put in the oven and dried at 110 °C for an hour until the quantity was constant. Then the dried sludge was milled in the mortar and screened by 5 mesh screen and 10 mesh screen to 2~4 mm size for preparation.

### The preparations of alkaline activated catalysts

Activated sludge was prepared as catalyst by immersing in activators (LiOH solution) with different concentration gradients (5%, 10%, 15%, 20%) for 2 hours. And the optimum concentration of activator was selected based on the denitration efficiency of the catalyst. Subsequently, the modified sludge was prepared under different activation time gradients (2 h, 4 h, 6 h) based on the optimal concentration. And the optimum activation time of the catalyst was selected based on the denitration efficiency of the catalyst.

### Preparation of sludge-supported catalyst

The modified sludge under optimal activation conditions loaded different loading mass (1%, 1.5%, 2%, 4%) of Mn(NO_3_)_2_ by constant volume impregnation method. The optimal loading mass of the catalyst was selected and evaluated from the SCO catalyst testing platform. It was calcined in the muffle furnace and the temperature of the muffle furnace was set to 450 °C. Sludge-supported catalysts were prepared by calcining at different calcination time gradients (2 h, 3 h, 4 h, and 5 h). The optimal calcination time of the catalyst was selected and evaluated from the SCO catalyst testing platform. Secondly, preparation of sludge-supported catalyst under different calcination temperature gradients (350 °C, 450 °C, 550 °C) was based on the optimal calcination time. The optimal calcination temperature was selected and evaluated from SCO catalyst testing platform.

### Catalyst evaluation device

**Figure 1:**
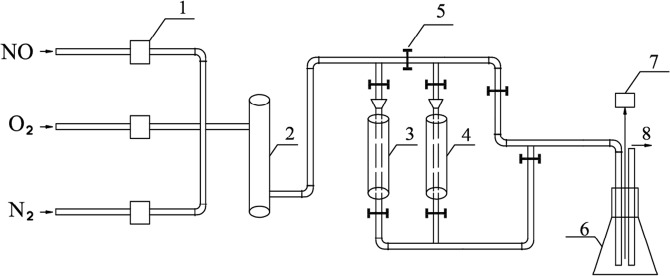
Experimental device of catalyst activity evaluation: 1, flowmeter; 2, gas mixing bottle; 3, reaction tower A; 4, reaction tower B; 5, valve control; 6, testing air bottle; 7, flue gas analyzer; 8, gaseous emission.

The experimental process diagram of catalyst activity evaluation is shown in Fig. 1.

The device is to evaluate the activity of catalyst prepared by different methods by simulating the flue gas. The simulated total gas flow rate is 1000 mL/min; the NO flow rate is 20 mL/min (content 600 ppm); the O_2_ flow rate is 60 mL/min (content 6%); the rest is filled with N_2_. The amount of catalyst used in the reaction is 3 g. These catalysts can be deposited into a reaction layer with a height of two centimeters in the reaction tower, in order to make the simulated flue gas and catalyst contact completely.

## Results and Discussion

### Effect on different activation concentration

**Figure 2:**
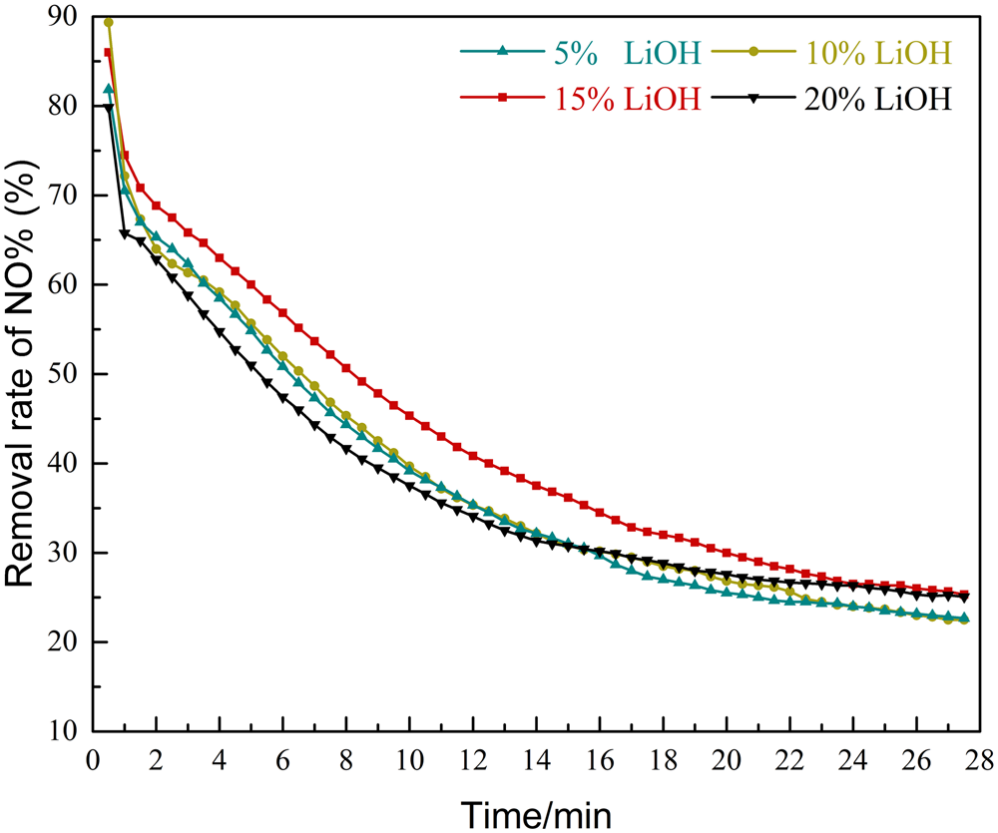
Influence of the sludge with different activation concentration of LiOH on NO removal efficiency.

The experimental results are presented in Fig. 2. The activation sludge with a mass concentration of 15% LiOH has the best removal effect on NO. As observed, in the reaction time of 2–17 min, the removal rate of NO in sludge activated by LiOH with 15% mass concentration was significantly higher than others. The removal rate of NO would be reduced if the concentration of activator is too high or too low. Several literatures have reported that results are consistent with this study, offering the justification that effective active sites cannot be formed on the sludge surface while the LiOH concentration is too low, and leading to a not obvious NO oxidation reaction. However, when the concentration of LiOH is too high, not only the activation of alkali reach saturation, but the excess alkali will destroy the active sites that have been formed^6,7^. Because the effect of the different concentration of the activator on the removal rate of NO was elucidated, the optimal mass concentration of activator LiOH is 15%.

### Effect on different activation time

**Figure 3:**
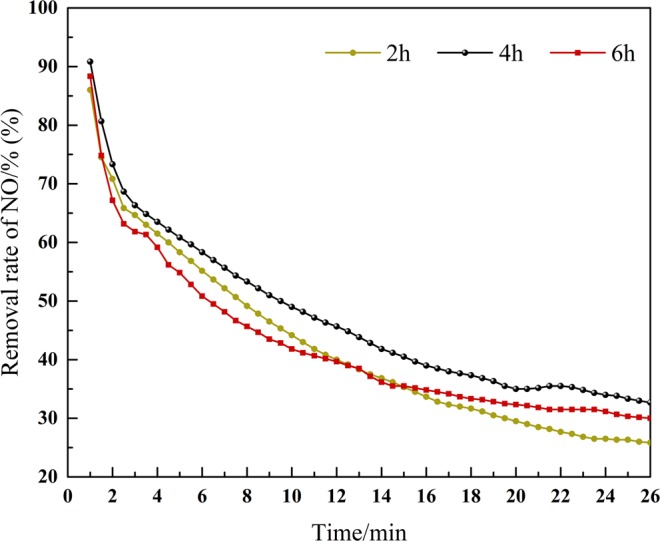
Influence of the sludge with different activation time of LiOH on NO removal efficiency.

As shown in Fig. 3, the sludge activated with activation time of 4 hours has the best removal effect on NO and that of 6 hours is the worst. Obviously, in the reaction time of 10–20 min, the catalyst with activation time of 4 hours has high rate of NO removal and persistence compared with 2 hours and 6 hours of activation. Regarding the influence of activation time, numerous studies agree with the results obtained in this study^8,9^. When the activation time of alkali is too short, LiOH cannot fully react with sludge, and there are few effective pore structures formed, so that the ideal activation effect cannot be achieved and sufficient active sites cannot be formed. With the increase of activation time, the activation efficiency and activation degree gradually become larger and deeper^10^. As a result, more and more effective holes are formed, and the denitration effect is enhanced gradually. When the activation time is too long, LiOH will destroy the micropore structure that has been formed before and reduce the number of active sites. Therefore, the efficiency of denitration decreases significantly. To sum up, the optimal activation time is 4 hours.

### Effect on different loading mass

**Figure 4:**
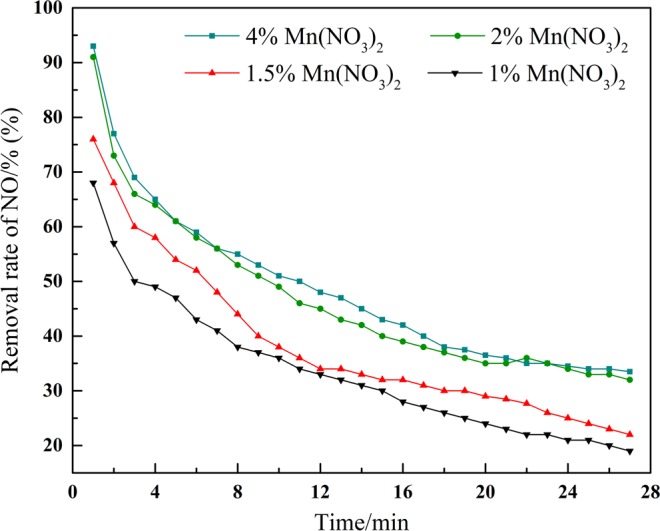
Influence of the sludge with different loading mass of Mn(NO_3_)_2_ on NO removal efficiency.

When the loading mass is small, the metal oxide cannot be evenly distributed on the surface of the catalyst, so that the denitration effect is not obvious. With the increase of the loading mass, the metal oxide is uniformly distributed on the catalyst surface, and the denitration effect is obvious. When the loading mass continues to increase, it will lead to the accumulation of active ingredients on the surface of the catalyst and fill in the hole structure of the catalyst, which will eventually lead to the destruction of the hole structure of the catalyst. It can be seen from the Fig. 4, When the loading mass is more than 2%, the increase of the removal rate of NO is obviously smaller. It is concluded that the optimum loading mass is 2%.

### Effect on different calcination time

**Figure 5:**
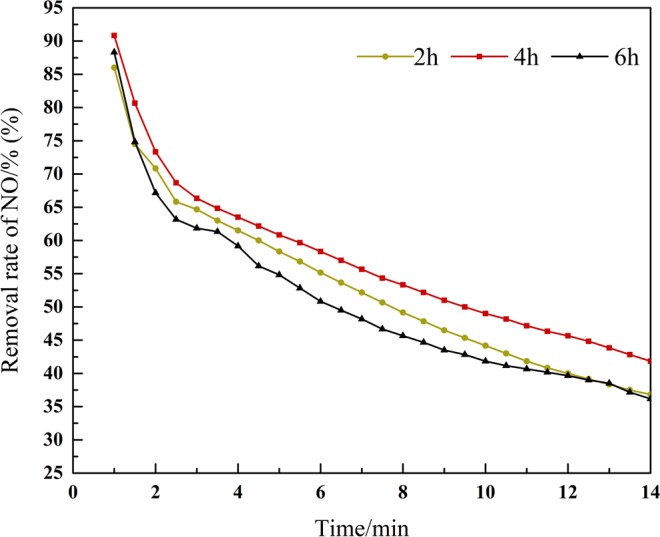
Influence of different calcination time of muffle furnace on NO removal efficiency.

The experimental results are presented in Fig. 5. It serves to show that MnOx-based catalyst with calcination time of 4 hours has the best removal effect on NO and that of 6 hours is the worst. Analysis of the reasons are as follows. When the calcination time is too short, the loaded Mn(NO_3_)_2_ is not completely burnt into the MnOx crystal type and the effective adsorption sites is not formed. On the other hand, when the calcination time is too long, the removal rate of NO is also reduced. The sintering of metal oxides on catalyst surface results in a significant reduction of active sites on catalyst surface. Reducing the rate of NO_2_ desorption and adsorption rate of NO so that the oxidation reaction is hindered^11–13^. Therefore, the optimal calcination time of the MnOx-based catalyst in the muffle furnace is 4 hours.

### Effect on different calcination temperature

**Figure 6:**
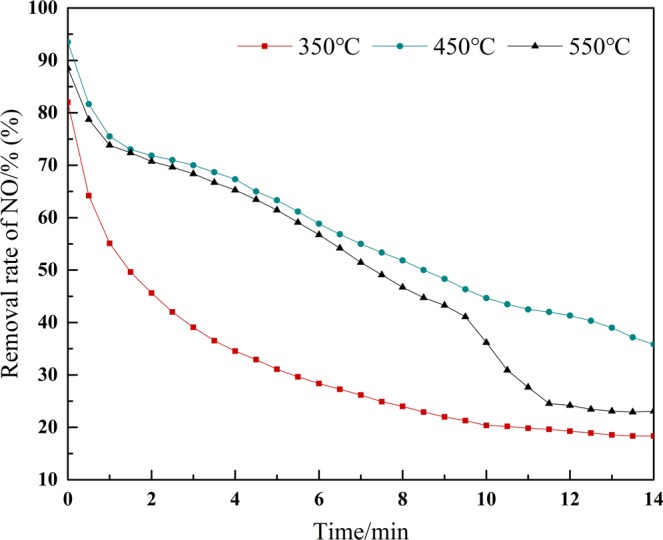
Influence of different calcination temperature of muffle furnace on NO removal efficiency.

Figure 6 demonstrates that the MnOx-based catalyst with calcination temperature of 450 °C has high rate of NO removal compared with 350 °C and 550 °C of calcination. When the calcination temperature is too low, the loaded Mn(NO_3_)_2_ is not completely burnt into the MnO_2_ crystal type and the effective adsorption sites is not formed. On the other hand, when the calcination temperature is too high, The sintering of metal oxides and the dispersion of MnO_2_ decreases to reduce the removal rate of NO^14–16^. Finally, the optimal calcination temperature is 450 °C.

### Infrared spectral characterization

**Figure 7:**
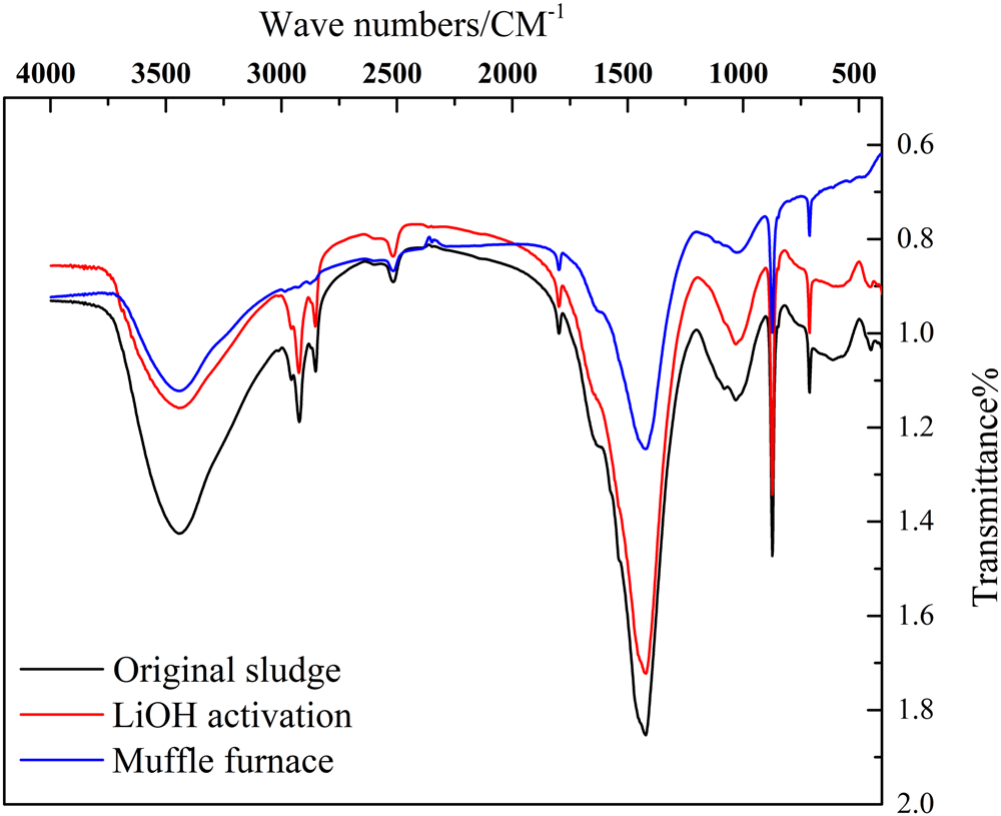
Infrared spectrogram of different catalysts.

The stretching vibrational absorption peak of −NO, −NO_2_ and −CH_2_ is at 874 cm^−1^, 1440 cm^−1^ and 2921 cm^−1^ respectively in the figures. All three groups come from organic matter in sludge. It can be seen from Fig. 7, in the peak of stretching vibrational absorption of 874 cm^−1^, 1440 cm^−1^ and 2921 cm^−1^, LiOH activated sludge and MnOx-based sludge all weakens compared with the original sludge. The original sludge consumes massive organic matters through alkaline activation. Since the calcinated sludge continuously consumes the organic matters of sludge, the stretching vibration of these three functional groups weakens consistently.

Because of the decrease of the functional groups of −NO, −NO_2_ and −CH_2_ in the sludge surface in Fig. 7, larger specific surface area and porosity of sludge are made. It is helpful for subsequently loading the active ingredient. In the Fig. 7, the continuous decrease of the functional groups of −NO, −NO_2_ and −CH_2_ in the sludge surface could make the adsorption property of catalyst better. It is more beneficial to the oxidation of NO to higher the NOx removal efficiency^17,18^.

Untreated soybean oil sludge is used as catalyst to remove NO, which is adsorbed on the sludge surface and reacted with O_2_ to form NO_2_. There are a great number of −OH on the surface of original sludge, which combines with NO_2_ to cause a not good desorption of NO_2_. The reactive sites of original sludge is occupied by NO_2_ and it makes the denitration rate lower. From these two figures it can be observed that the peak of stretching vibrational absorption of −OH is 2570 cm^−1^. The continuous decrease of the functional group of −OH is achieved after LiOH activation and calcination^19^. −OH is a highly activated oxygen-containing functional group. The decrease of −OH not only provides more active sites for subsequently loading the metal oxide, but also decreases the re-adsorption on NO_2_ to make a better desorption of NO_2_^20^.

### XRD characterization

**Figure 8:**
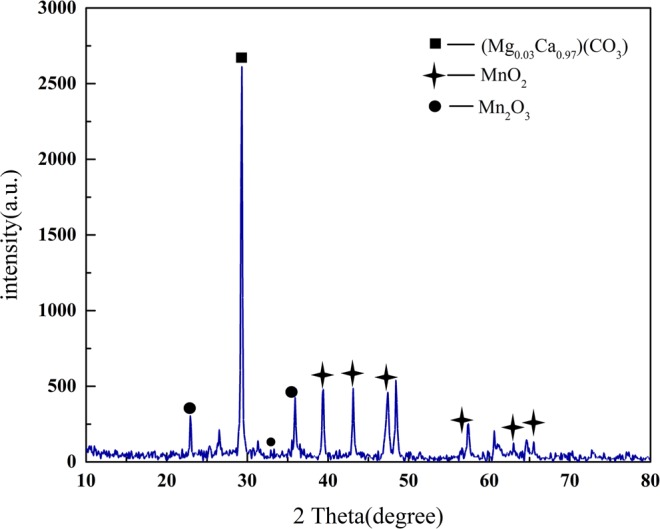
XRD spectrogram of MnOx-based sludge calcined by muffle furnace.

The spectrum of XRD test about loaded sludge after calcining in muffle furnace is shown in Fig. 8.

It can be seen from XRD spectrogram that characteristic peaks of MnO_2_ crystal appear at the angles θ of 39.93°, 43.52°, 47.71°, 56.90°, 63.25°and 65.70° (JCPDS No. 43–1455 and No. 44–0142). And characteristic peaks of Mn_2_O_3_ crystal form appear at the angles θ of 23.13°, 32.92°and 35.68° (JCPDS No. 41–1442). The main oxide crystals of Mn in loaded catalyst after calcining in muffle furnace are MnO_2_ and Mn_2_O_3_. The oxide of Mn provides the precondition for the catalyst to choose NO.

### XPS characterization

**Figure 9:**
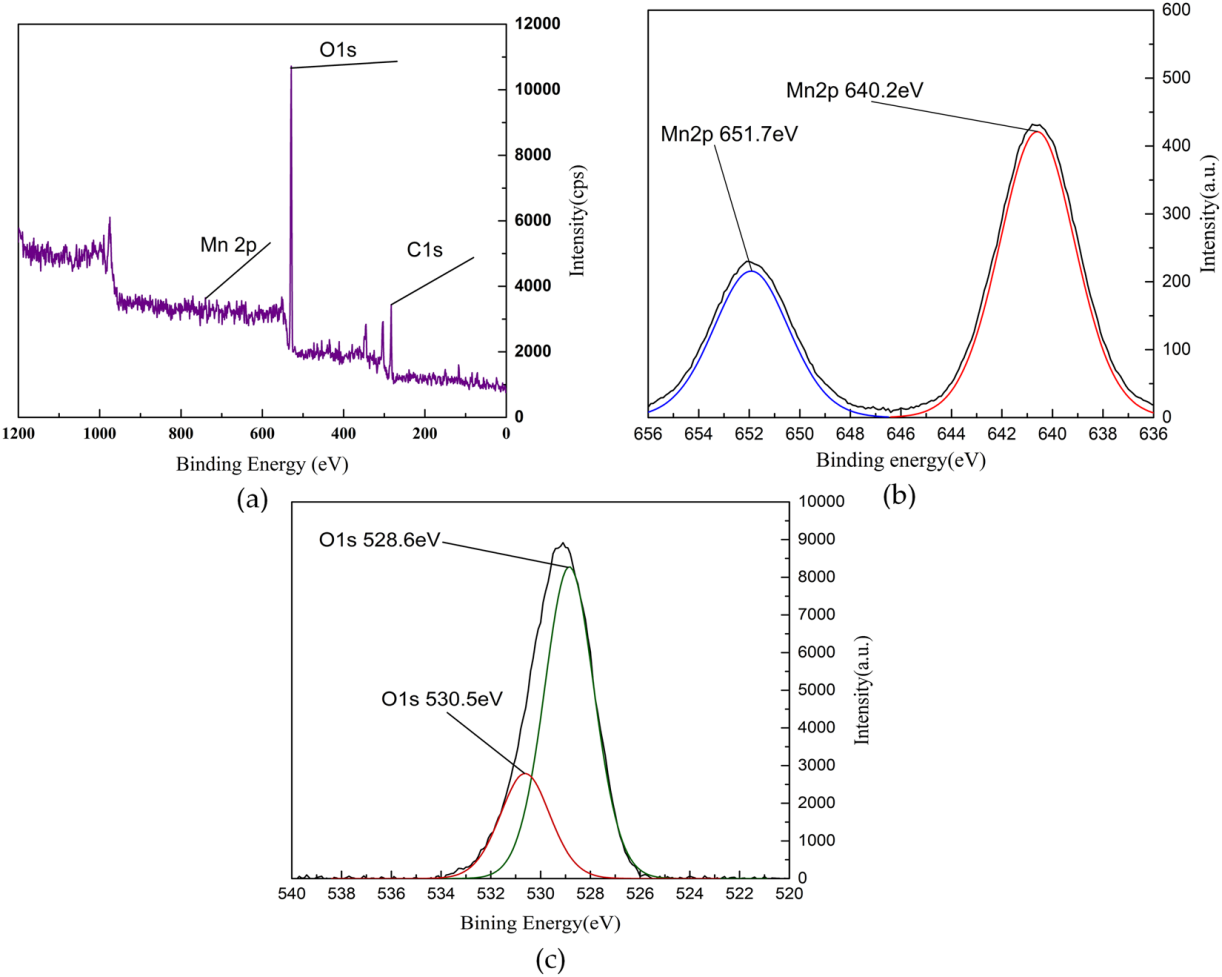
XPS spectrogram of MnOx-based sludge (not involved in the denitration reaction). (**a**) Full scan spectra of MnOx; (**b**) Mn 2 P spectra of MnOx; (**c**) O 1 s spectra of MnOx.

**Figure 10:**
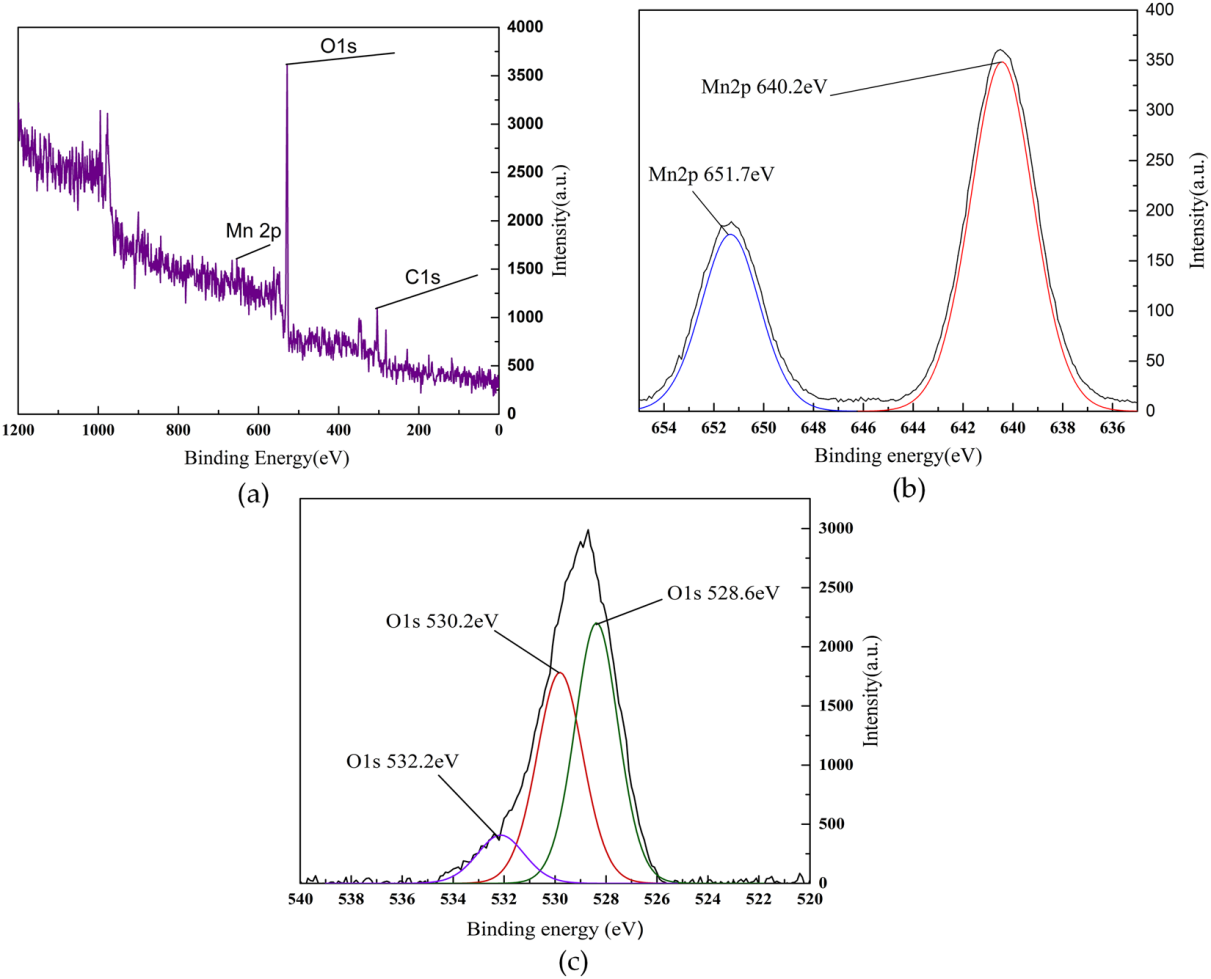
XPS spectrogram of MnOx-based sludge (involved in the denitration reaction). (**a**) Full scan spectra of MnOx; (**b**) Mn 2 P spectra of MnOx; (**c**) O 1 s spectra of MnOx.

The spectrum of XPS test about loaded sludge calcined in muffle furnace before and after denitration reaction is shown in Figs 9 and 10.

Figures 9 and 10 show that MnOx-based catalyst contains mainly oxygen, carbon and manganese elements. It can be seen from the spectrogram of Mn that there are two peaks of Mn on the surface of catalyst, which are Mn2p3/2 and Mn2p1/2. Two kinds of manganese oxides with different valence states in catalyst are explained. The binding energy of Mn2p3/2 peak is 640.2 eV, whose substance is MnO_2_. And the binding energy of Mn2p1/2 peak is 651.7 eV, whose substance is Mn_2_O_3_. The main oxide crystals of Mn in loaded catalyst calcined by muffle furnace are MnO_2_ and Mn_2_O_3_.

From the figures it can be concluded that, the intensity peaks of binding Energy of 528.6 eV and 530.2 eV are lattice oxygen (O^2−^) in different chemical environments. The intensity peak of binding energy of 532.2 eV is chemisorbed oxygen (O_2_^2−^, O^−^). The charge imbalance of catalyst surface could be caused by Mn^3+^ and Mn^4+^ firstly. The hole in the electron track of Mn could be caused secondly. The various unsaturated chemical bonds could be formed lastly^21–23^. These above conditions are beneficial to generate the chemisorbed oxygen, which has strong mobility and plays an important role in influencing catalyst performances and activation.

When the catalyst is involved in the catalytic reaction in SCO, the chemical environment of lattice oxygen is changed after a series of redox reactions to change the intensity of peak. The oxygen in the air is not completely decomposed into O at high temperatures^24–26^. When the simulated flue gas containing O and O_2_ passes through the catalyst, the electron in the e_g_ orbit in Mn^3+^ are transferred to O and O_2_, some of them are converted into the chemisorbed oxygen. At this time, Mn^3+^ transforms to Mn^4+^ due to the loss of the electron, the number of holes increases^27–30^. And the chemisorbed oxygen, which oxidizes NO to the target product NO_2_ can be adsorbed on the surface of catalysts. The partially adsorbed chemisorbed oxygen can be converted to O^2−^ by getting electrons^31,32^. Because the Mn^3+^ and Mn^4+^ loaded in sludge are separated and the eg orbit in Mn^4+^ are empty, the electron in eg orbits can be transited from Mn^3+^ to Mn^4+^ through the double exchange of O^2−^ ^33^. Besides, the double exchange between Mn^3+^ and Mn^4+^ can also cause the charge imbalance on the surface of the catalyst so that the number of the holes increases to promote the adsorption of chemisorbed oxygen and enhance the denitration efficiency^34,35^.

### SEM characterization

**Figure 11:**
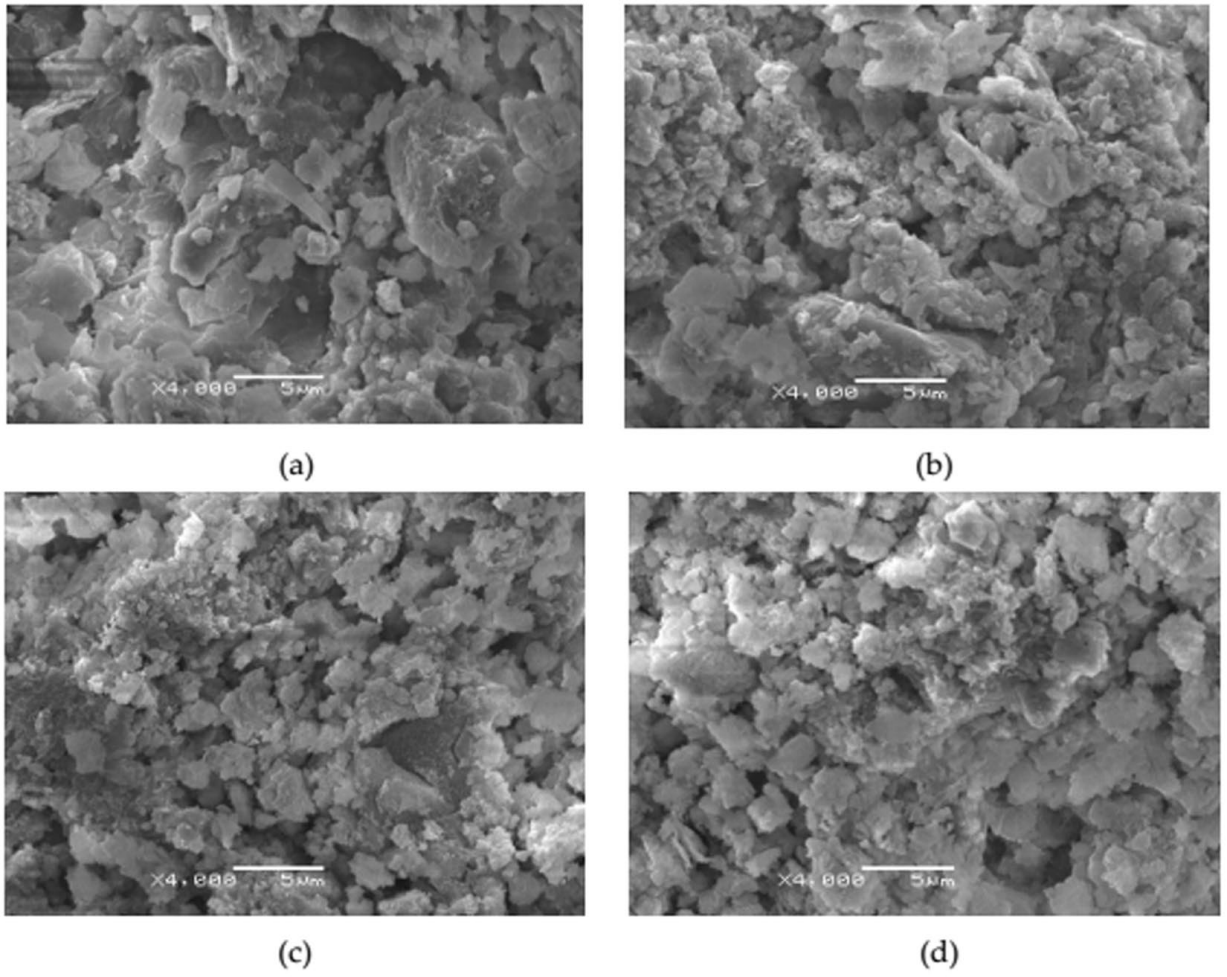
Scanning electron microscope image of catalyst. (**a**) Original sludge; (**b**) alkaline activated sludge; **(c**) MnOx-based sludge (involved in the denitration reaction); (**d**) MnOx-based sludge (not involved in the denitration reaction).

The amplification time of original sludge, alkaline activated sludge and loaded sludge calcined in muffle furnace before and after denitration reaction are 4000 times. The result is shown in Fig. 11.

There are obvious crystals on the surface of loaded sludge after calcined by muffle furnace from Fig. 11. As explained in Fig. 10 above, these crystals are manganese oxides. Comparing with Fig. 11(c,d), the increase in particle size after sludge reaction is due to the formation of new reaction products on the sludge surface after reaction.

## Conclusion

This study originally proposed to use soybean oil sludge as the carrier of the catalyst and carry out the alkaline activation treatment of the sludge then the impregnation method was applied to prepare the MnOx-based sludge. On this basis, MnOx-based sludge was calcined by muffle furnace. A series of activated sludge and MnOx-based catalysts were prepared. The catalyst activity was evaluated in the simulated SCO denitration process. The effects of activation conditions and calcination conditions on catalyst activity were investigated. Fourier transform infrared (FTIR), X-ray photoelectron spectroscopy (XPS), X-ray diffraction (XRD) and scanning electron microscopy (SEM) detection methods were used to characterize the activity of the sludge based denitration catalyst, and the structure and activity of the MnOx-based denitration catalyst was furtherly confirmed. The results show that:

1. The surface of the sludge catalyst obtained after the sludge activated by LiOH with a mass concentration of 15% for 4 hours has more alkali functional groups, making the catalyst for denitration of sludge the best.
2. The MnOx-based catalyst calcined in the muffle furnace with calcination temperature of 450 °C for 4 hours has obvious denitration efficiency.

## Supplementary information

Individual figures from Figure 9 and Figure 10
