# Supplementary material for: Preparation of MnOx Supported LiOH Activated Soybean Oil Sludge Catalyst and Its Analysis in Denitration Mechanism of Selective Catalytic Oxidation (SCO)
Source: Sci Rep. 2019 Aug 12;9:11604. doi: 10.1038/s41598-019-47947-2 (PMC6690868; doi:10.1038/s41598-019-47947-2)
Supplement: Supplementary file 1 — Individual figures from Figure 9 and Figure 10 [file 41598_2019_47947_MOESM1_ESM.docx]

Preparation of MnOx Supported LiOH Activated Soybean Oil Sludge Catalyst and Its Analysis in Denitration Mechanism of Selective Catalytic Oxidation (SCO)

Zhang Lei^*12^, Luo Min^1^, Kong Tingting^3^ , Zhang Lei^4^, He Huibin^1^, Jia Yang^1^, Yang Chao^1^, Wu Yan^5^, Li mengting^6^

1. *Xi’an University of Science and Technology, Xi’an, 710054, China*

*2.* *Key Laboratory of Coal Resources Exploration and Comprehensive Utilization，Ministry of Land and Resources Xi’an, 710021, China*

3. *Xi’an Shiyou University, Xi’an, 710065, China*

4. *China National Heavy Machinery Research Institute, Xi’an, 710032, China*

5. *Key Laboratory of Coal Resources Exploration and Comprehensive Utilization, Ministry of Land and Resources, Xi’an, 710054, China*

6. *Neijiang Normal University, Neijiang, 641100, China*

** Correspondence: Zhang Lei:* [*136750178@qq.com*](mailto:136750178@qq.com)


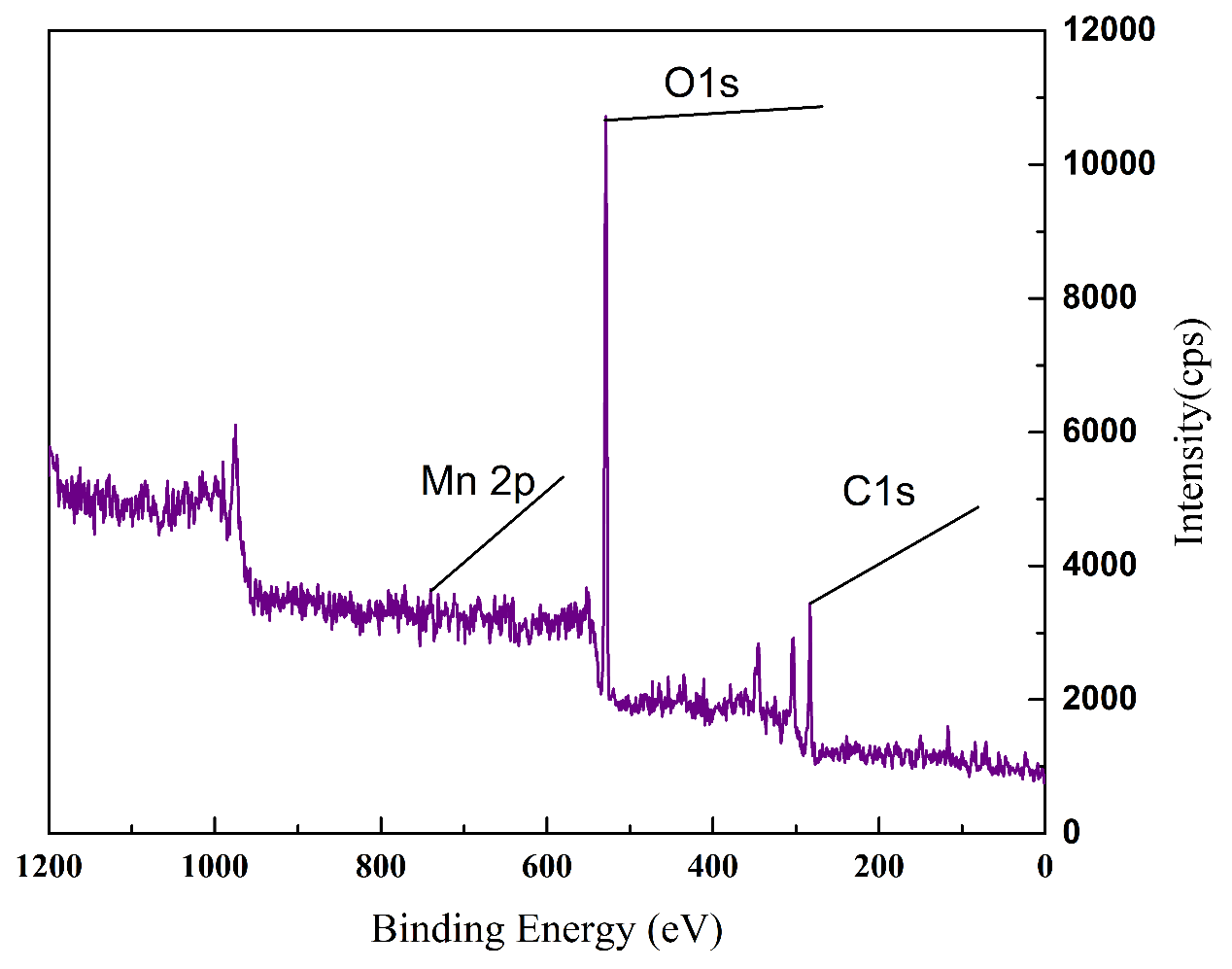


Figure 9(a)


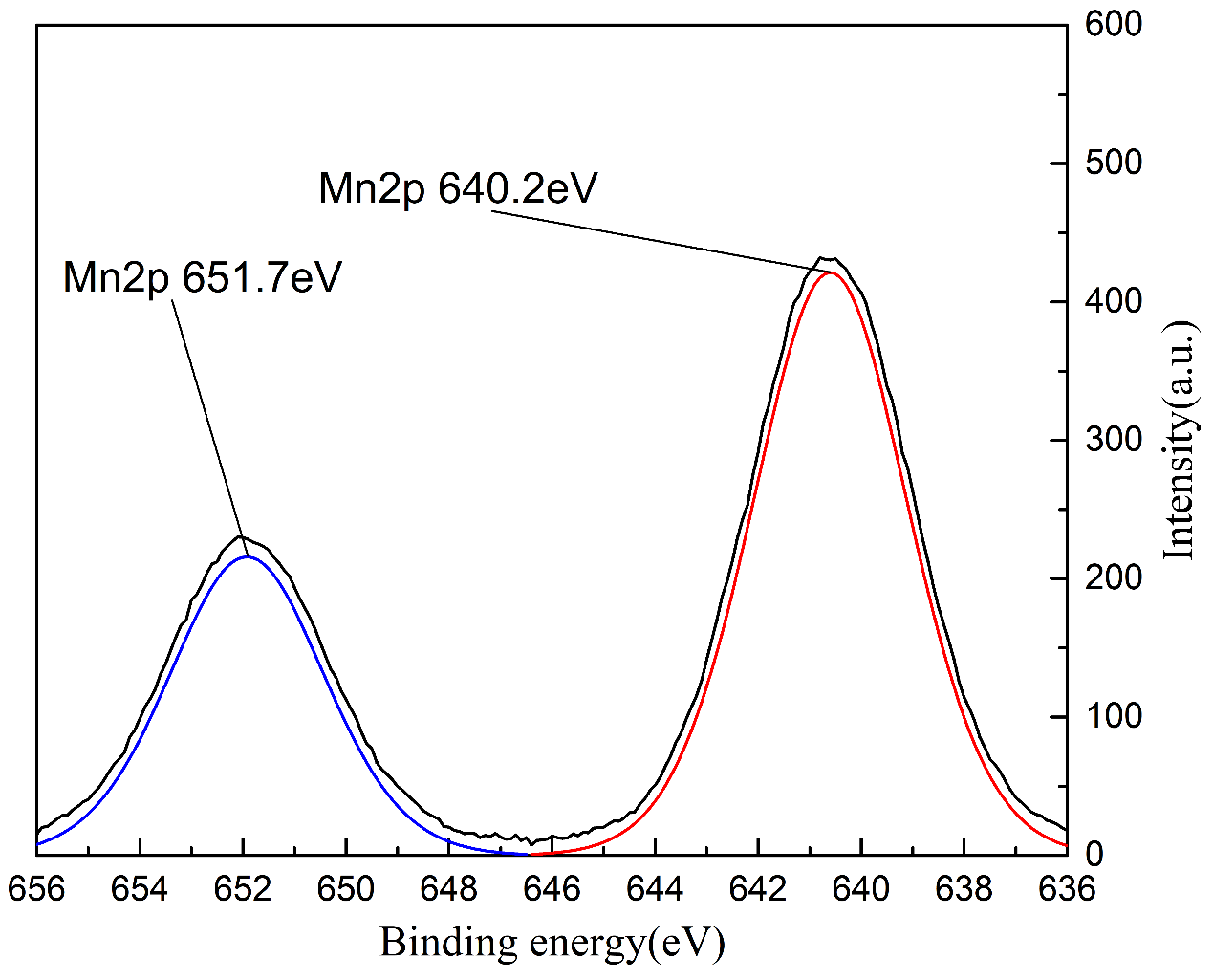


Figure 9(b)


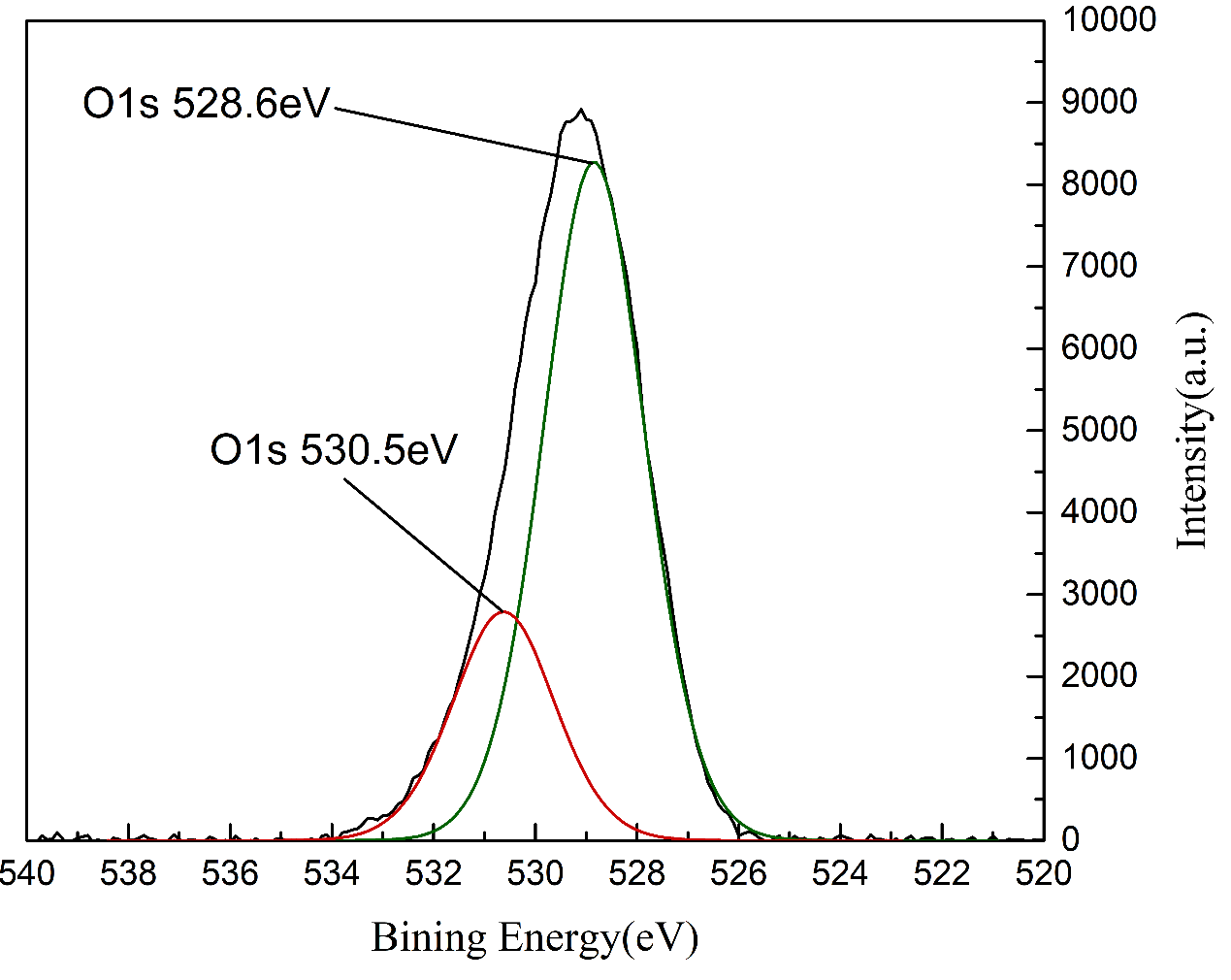


Figure 9(c)


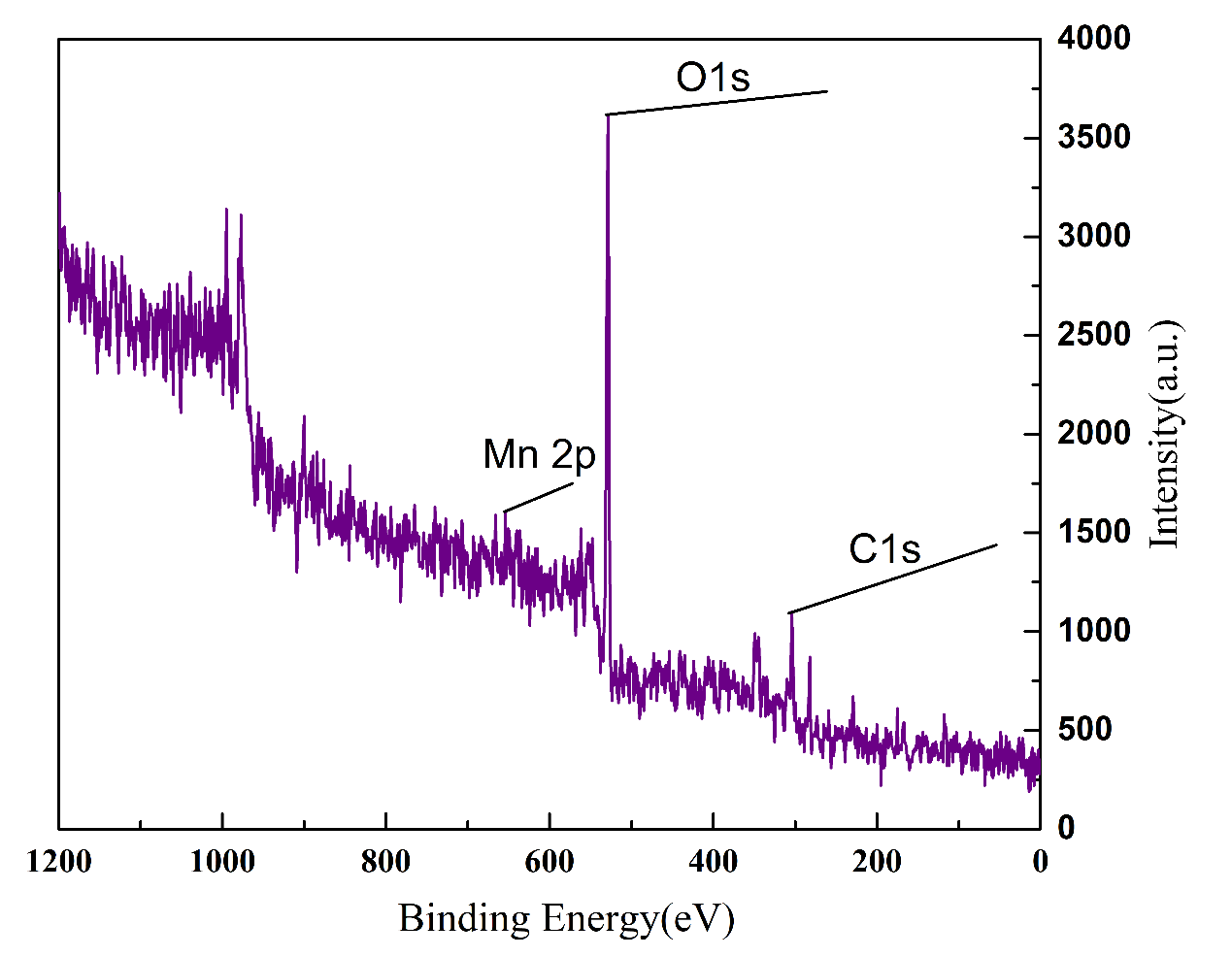


Figure 10(a)


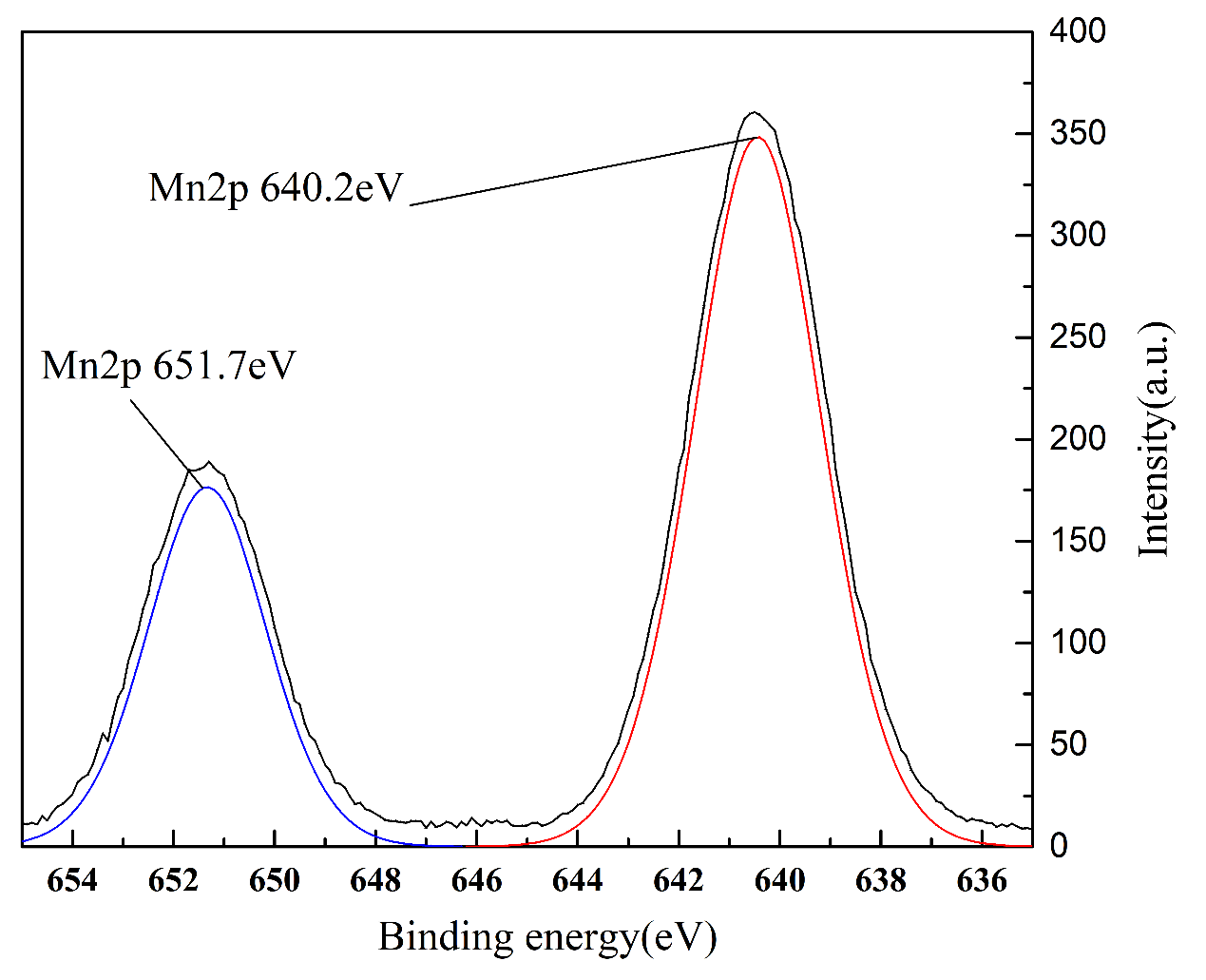


Figure 10(b)


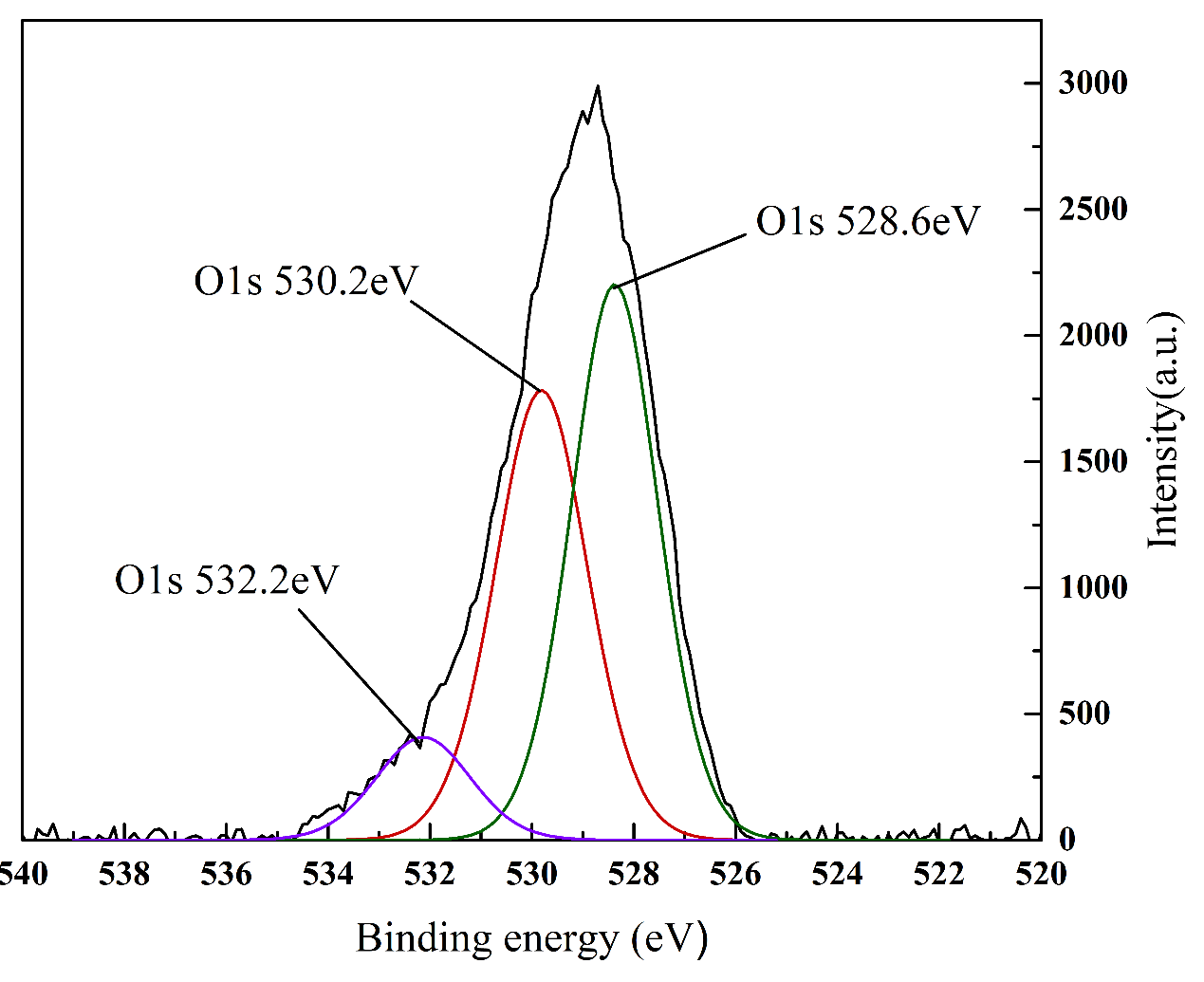


Figure 10(c)
